# Increased risk of HIV and other drug-related harms associated with injecting in public places: national bio-behavioural survey of people who inject drugs

**DOI:** 10.1016/j.drugpo.2020.102663

**Published:** 2020-03

**Authors:** Kirsten M.A. Trayner, Andrew McAuley, Norah E. Palmateer, David J. Goldberg, Samantha J. Shepherd, Rory N. Gunson, Emily J. Tweed, Saket Priyadarshi, Catriona Milosevic, Sharon J. Hutchinson

**Affiliations:** a School of Health and Life Sciences, Glasgow Caledonian University, Glasgow, UK; b Health Protection Scotland, Glasgow, UK; c West of Scotland Specialist Virology Centre, Glasgow, UK; d MRC/CSO Social and Public Health Sciences Unit, University of Glasgow, Glasgow, UK; e NHS Greater Glasgow and Clyde Addictions Services, Glasgow, UK; f Public Health Protection Unit, NHS Greater Glasgow and Clyde, Glasgow, UK

**Keywords:** Public injecting, Drug-related harms, HIV, Harm reduction, People who inject drugs

## Abstract

**Background:**

Whilst injecting drugs in public places is considered a proxy for high risk behaviour among people who inject drugs (PWID), studies quantifying its relationship with multiple drug-related harms are lacking and none have examined this in the context of an ongoing HIV outbreak (located in Glasgow, Scotland). We aimed to: 1) estimate the prevalence of public injecting in Scotland and associated risk factors; and 2) estimate the association between public injecting and HIV, current HCV, overdose, and skin and soft tissue infections (SSTI).

**Methods:**

Cross-sectional, bio-behavioural survey (including dried blood spot testing to determine HIV and HCV infection) of 1469 current PWID (injected in last 6 months) recruited by independent interviewers from 139 harm reduction services across Scotland during 2017–18. Primary outcomes were: injecting in a public place (yes/no); HIV infection; current HCV infection; self-reported overdose in the last year (yes/no) and SSTI the last year (yes/no). Multi-variable logistic regression was used to determine factors associated with public injecting and to estimate the association between public injecting and drug-related harms (HIV, current HCV, overdose and SSTI).

**Results:**

Prevalence of public injecting was 16% overall in Scotland and 47% in Glasgow city centre. Factors associated with increased odds of public injecting were: recruitment in Glasgow city centre (aOR=5.45, 95% CI 3.48–8.54, *p*<0.001), homelessness (aOR=3.68, 95% CI 2.61–5.19, *p*<0.001), high alcohol consumption (aOR=2.42, 95% CI 1.69–3.44, *p*<0.001), high injection frequency (≥4 per day) (aOR=3.16, 95% CI 1.93–5.18, *p*<0.001) and cocaine injecting (aOR=1.46, 95% CI 1.00 to 2.13, *p* = 0.046). Odds were lower for those receiving opiate substitution therapy (OST) (aOR=0.37, 95% CI 0.24 to 0.56, *p*<0.001) and older age (per year increase) (aOR=0.97, 95% CI 0.95 to 0.99, *p* = 0.013). Public injecting was associated with an increased risk of HIV infection (aOR=2.11, 95% CI 1.13–3.92, *p* = 0.019), current HCV infection (aOR=1.49, 95% CI 1.01–2.19, *p* = 0.043), overdose (aOR=1.59, 95% CI 1.27–2.01, *p*<0.001) and SSTI (aOR=1.42, 95% CI 1.17–1.73, *p*<0.001).

**Conclusions:**

These findings highlight the need to address the additional harms observed among people who inject in public places and provide evidence to inform proposals in the UK and elsewhere to introduce facilities that offer safer drug consumption environments.

## Introduction

Globally, there are approximately 15.6 million people who inject drugs (PWID) (Degenhardt et al., 2017) who are at risk of a range of harms including blood-borne viruses (BBVs), skin and soft tissue infections (SSTI) (Gordon & Lowy, 2005) and fatal and non-fatal overdose (Degenhardt et al., 2011; Mathers et al., 2013). These harms are influenced by an individual's injecting environment (or their “risk environment”), which relates to physical, social, economic and political factors at a macro- and micro-environmental level (Rhodes, 2002, 2009). An understanding of the current risk environments faced by PWID is important to improve and tailor harm reduction services and healthcare provision.

Public injecting (defined as the injection of drugs in an area accessible to the general public, such as alleyways, public toilets, stairwells, etc.) (Hunt, Lloyd, Kimber & Tompkins, 2007; Parkin & Coomber, 2009; Tweed, Rodgers, Priyadarshi & Crighton, 2018) concerns an individual's physical microenvironment (Rhodes et al., 2006). Ethnographic research has demonstrated a number of features of these environments that increase risk, such as lack of a hygienic area, including access to clean water to wash hands and injecting sites, to facilitate good injecting hygiene. Additionally, poor lighting and hurried injections increase the risk of missed hits (Small, Rhodes, Wood & Kerr, 2007). These environmental conditions increase the risk of abscesses, SSTI, and other injection site-related issues (Salmon et al., 2009). Given its public nature, PWID injecting in public places are also more likely to experience arrest and incarceration (DeBeck et al., 2009; Hunter et al., 2018; Ickowicz et al., 2017; Marshall, Kerr, Qi, Montaner & Wood, 2010; Small et al., 2007). Fear of assault and being discovered by the public or the police encourages rushed higher risk injections where PWID are less likely to follow safer injection practices (Dovey, Fitzgerald & Choi, 2001; Small et al., 2007). These conditions increase the risk of fatal and non-fatal overdose, which has been consistently demonstrated in the literature (Darke, Kaye & Ross, 2001; Hunter et al., 2018; Sutter, Curtis & Frost, 2019; Vallance et al., 2018; Wallace, Kennedy, Kerr & Pauly, 2019).

PWID injecting in public places are often the most vulnerable and marginalised members of society, with homelessness and socio-economic deprivation being the strongest predictors of public injecting (Havinga, Van der Velden, De Gee & Van der Poel, 2014; Hunt et al., 2007; Ickowicz et al., 2017; Marshall et al., 2010; McKnight et al., 2007; Navarro & Leonard, 2004; Vallance et al., 2018). Intertwined with the precarious context associated with homelessness, public injecting has also been associated with a higher severity of addiction with complex health and social needs, which leads to higher intensity and higher risk drug use (Navarro & Leonard, 2004; Vallance et al., 2018). Furthermore, the social network dynamics of PWID who inject in public places can normalise high risk injection behaviour (Tobin, Davey-Rothwell & Latkin, 2010; Weeks et al., 2001); PWID injecting in public are more likely to purchase, share (through the practices of “frontloading” and “backloading”) and inject drugs in groups (Navarro & Leonard, 2004). This increases the likelihood of sharing injecting equipment, which has also consistently been associated with public injecting (Hunter et al., 2018; Marshall et al., 2010; Mazhnaya, Tobin & Owczarzak, 2018; McKnight et al., 2007; Navarro & Leonard, 2004; Vallance et al., 2018; Wood et al., 2001).

While research has shown that public injecting is related to a number of risk factors (such as the sharing of injecting equipment) which increase the risk of BBV transmission, evidence on the direct association between public injecting and serological evidence of BBV infection is limited. Previous research has demonstrated a significant association between public injecting and ever exposure (antibody positive) to hepatitis C virus (HCV) through serological testing (Boodram, Golub & Ouellet, 2010; Maher, Chant, Jalaludin & Sargent, 2004). Only one previous study conducted during 1988–91 demonstrated injecting in outdoor settings or abandoned buildings to be a significant predictor of HIV seroconversion in low prevalence cities in the United States (Friedman, Jose, Deren, Des Jarlais & Neaigus, 1995). However, no study to our knowledge has demonstrated an association between serological evidence of HIV and public injecting, in the era of highly active anti-retroviral therapy (HAART), opioid substitution therapy (OST) and needle and syringe provision. In addition, we are not aware of any single study which considers the association between multiple drug-related harms (including serological evidence of current HCV/HIV, overdose and SSTI), and public injecting. The aims of this study were thus to estimate: 1) the extent of public injecting in Scotland and associated individual and demographic/environmental risk factors, and 2) the association between public injecting and drug-related harms (HIV, current HCV, overdose and SSTI). To the best of our knowledge, this is the largest study and only one at a national level to have considered this. Further, our focus on public injecting is particularly warranted in the context of the largest recorded outbreak of HIV among PWID in the UK in the last 30 years, identified in Glasgow (McAuley et al., 2019; Ragonnet-Cronin et al., 2018), in which public injecting has been described as a prominent feature among cases (Tweed et al., 2018).

## Methods

### Data source

This study utilised data collected in 2017–18 by the Needle Exchange Surveillance Initiative (NESI), a national cross-sectional bio-behavioural survey conducted every two years in Scotland since 2008 (Health Protection Scotland, 2019a). NESI aims to monitor the prevalence of BBVs and injecting risk behaviours among PWID. After providing written informed consent, participants complete an interviewer-led questionnaire and provide a dried blood spot (DBS) sample, which is tested anonymously for BBV markers. Questions related to public injecting were added to the most recent survey conducted between July 2017 and October 2018; the full questionnaire is available at: https://www.hps.scot.nhs.uk/web-resources-container/needle-exchange-surveillance-initiative-nesi-2008-09-to-2017-18/.

PWID were recruited by independent interviewers at 139 sites providing harm reduction services across Scotland (approximately half of the total nationally). Sites were chosen to be broadly representative of the 11 regional administrative health areas (National Health Service (NHS) Boards) in mainland Scotland. Inclusion criteria for participants are: a) having injected drugs on at least one occasion (either recently (last 6 months) or in the past) and b) no previous participation in the current survey. We here considered current PWID, defined as those who had injected in the previous six 6 months (relating to approximately 70% of the total sample). A total of 1469 PWID were available for analysis, after the removal of: participants who had not injected in the previous 6 months (*n* = 675) and duplicate participants (identified using initials, gender and date of birth) (*n* = 61).

### Outcomes

The primary outcomes of interest were: injecting in a public place in the last 6 months (yes/no); HIV infection; current HCV infection; overdose in the last year (yes/no) and SSTI the last year (yes/no). An individual was considered to have injected in public if they had reported injecting in a public toilet, car park, stairwell/close (communal hall and stairway in residential building), outdoors (park, street, etc.) and squat/abandoned house. Public injecting in the last 6 months was used as both an outcome and exposure variable. HIV and HCV infection were confirmed by laboratory DBS tests (Health Protection Scotland, 2019a). HIV was detected using the Abbott Architect antigen/antibody combination assay. HCV antibody was detected using the Abbott Architect and HCV RNA was extracted and amplified using an in-house real-time PCR method (Bennett et al., 2012). Samples which tested both antibody and PCR positive were assumed to represent those with current HCV infection.

We assessed outcomes according to relevant demographic/environmental and individual risk factors. Exposure variables refer to the last 6 months unless stated otherwise. Demographic/environmental exposures of interest included: gender (male/female), age, homelessness (yes/no), number of times in prison since first injected drugs (≤5 times)/5> times), recruitment region (Glasgow city centre/Greater Glasgow and Clyde (excluding city centre)/rest of Scotland), received opioid substitution therapy (OST) (yes/no), prescribed naloxone in the last 12 months (yes/no), arrested for drug offenses (yes/no) and needle and syringe coverage per injecting episode (<100%/100%+). Individual risk factors of interest included: injected heroin (yes/no), injected cocaine (yes/no), injected heroin and cocaine (yes/no), injecting heroin and cocaine at the same time (also known as ‘snowballing’) (yes/no), shared needles/syringes (yes/no), years since first injection (<10 years/>10+ years), injection frequency (≤4 times per day)/>4 times per day), reused needle/syringe (yes/no), unprotected sex (yes/no) and alcohol consumption (>14 units per week) (in line with UK Government guidance) in the last 12 months (yes/no).

### Analyses

The proportions of respondents having reported recent public injecting were initially compared with those who had not across demographics, risk behaviours, uptake of harm reduction services and harm variables using chi-squared tests of association. Unadjusted and adjusted logistic regression was subsequently used to estimate: 1) factors associated with public injecting, 2) the association between public injecting and HIV, current HCV, overdose and SSTI. Our modelling methodology was underpinned by the minimum criteria for sample size considerations in logistic regression, which states that there must be 10 events per independent variable included in the model (Peduzzi, Concato, Kemper, Holford & Feinstein, 1996). Furthermore, we used a significance level of *p*<0.05. To assess the potential of multicollinearity in our models, we computed a correlation matrix to ensure that none of the variables included were correlated with each other. All correlation coefficients had an r-value of < ± 0.50. Analysis was undertaken using Stata 13.

#### Factors associated with public injecting

We grouped potential risk factors for public injecting into ‘individual’ and ‘demographic/environmental’ categories (as stated above). The first model assessed demographic/environmental factors associated with public injecting and the second model assessed individual factors associated with public injecting (can be viewed in Appendix 1 and 2, respectively). Factors that were significant (*p*<0.05) in both the individual and demographic/environmental models were then entered into a combined model.

#### Harms (HIV, HCV, overdose and SSTI)

Four separate models were constructed, one for each drug-related harm. All models were adjusted for age and gender. Co-variates included in each model were based on known associations derived from the literature and only included if the co-variate was associated with both public injecting and the relevant harm. For the HIV model, homelessness and cocaine injecting were included (McAuley et al., 2019). The HCV model included homelessness, cocaine injecting, injection frequency and alcohol consumption. The overdose model additionally included homelessness, cocaine injecting, alcohol consumption and injection frequency (O'Halloran et al., 2017; Riley et al., 2016). The SSTI model included, homelessness, injecting heroin and cocaine at the same time and injection frequency (Brown & Ebright, 2002). Full models including co-variates can be viewed in the Appendix 3–6.

A multi-level framework was applied to each harms model to account for potential clustering across the 11 NHS Scotland board recruitment regions. To account for clustering of HIV cases in NHS Greater Glasgow and Clyde (NHS GGC) as a result of the ongoing HIV outbreak (and too few observations elsewhere), the HIV multi-level model framework was collapsed into NHS Greater Glasgow and Clyde versus rest of Scotland.

## Results

### Participant characteristics

Of the 1469 PWID, the majority were male (75%, *n* = 1095) and the mean age was 39.6 years (Table 1). Just over a quarter (27%, *n* = 401) had experienced recent homelessness and 37% (*n* = 542) had been in prison more than five times. The vast majority (92%, *n* = 1345) of the sample had injected heroin in the past six months and 31% (*n* = 452) reported injecting cocaine. The prevalence of HIV and current HCV infection was 3% (*n* = 42) and 32% (*n* = 402), respectively. Prevalence of overdose and SSTI in the last year was 18% (*n* = 264) and 28% (*n* = 401), respectively.

**Table 1:** Demographics, risk behaviours, uptake of harm reduction services and harms reported by 1469 PWID in Scotland during 2017–18, according to their self report of having injected in a public place in the previous 6 months.

	Total sample^a^^,^*N* (%)	PWID who reported public injecting^a^ (% of *N*)	χ^2^	P-value^b^
**Total (5 non-responses regarding public injecting)**	1469 (100)	240 (16)	N/A	N/A
***Demographics***				
**Gender (4 non-responses)**^c^				
Male	1095 (75)	196 (18)		
Female	366 (25)	43 (12)	7.6	0.006
**Age (mean)**	39.6	38.6	N/A	N/A
**Health board region**				
Glasgow city centre	219 (15)	102 (47)		
NHS Greater Glasgow and Clyde^d^	365 (25)	51 (14)	174.3	<0.001
Rest of Scotland e	880 (60)	87 (10)		
**Homeless in the last 6 months (2 non-responses)**				
Yes	401 (27)	148 (37)		
No	1061 (73)	91 (9)	170.8	<0.001
**Number of times in prison (13 non-responses)**				
5 or less	911 (63)	119 (13)		
More than 5	542 (37)	119 (22)	19.6	<0.001
**Arrested for drug offenses in the last 6 months (39 non-responses)**				
Yes	182 (13)	38 (21)		
No	1245 (87)	193 (16)	3.4	0.066
***Risk behaviours***				
**Injected heroin in the last 6 months (4 non-responses)**				
Yes	1345 (92)	221 (16)		
No	118 (8)	19 (16)	0.1	0.926
**Injected cocaine in the last 6 months (5 non-responses)**				
Yes	452 (31)	129 (28)		
No	1010 (69)	111 (11)	70.1	<0.001
**Injected heroin and cocaine in the last 6 months (5 non-responses)**				
Yes	337 (23)	108 (32)		
No	1125 (77)	132 (12)	78.0	<0.001
**Shared needles/syringes in last 6 months (14 non-responses)**				
Yes	141 (10)	34 (24)		
No	1311 (90)	201 (15)	7.2	0.007
**Years since first injection (16 non-responses)**				
<10 years	389 (27)	57 (15)		
10+ years	1060 (73)	182 (17)	1.3	0.253
**Average injection frequency in last 6 months (6 non-responses)**				
Low frequency (less than four times per day)	1345 (92)	186 (14)		
High frequency (4 or more times per day)	117 (8)	54 (46)	81.9	<0.001
**Reused needle/syringe re-use in last 6 months (16 non-responses)**				
Yes	848 (58)	167 (20)		
No	602 (42)	66 (11)	19.9	<0.001
**Unprotected sex in last 6 months**				
Yes	609 (42)	91 (15)		
No	733 (50)	128 (17)	1.5	0.214
Unknown/missing	122 (8)	21 (17)		
**Alcohol consumption (>14 units per week) in the last 12 months (16 non-responses)**				
Yes	317 (22)	100 (32)		
No	1133 (78)	137 (12)	68.6	<0.001
***Coverage of harm reduction interventions***				
**Received OST in the last 6 months (1 non-response)**				
Yes	1247 (85)	174 (14)		
No	217 (15)	66 (30)	36.5	<0.001
**Needle/syringe coverage per injecting episode in last 6 months**				
100%+	1016 (69)	149 (15)		
<100%	281 (19)	51 (18)	2.1	0.152
Unknown/missing	167 (11)	40 (24)		
**Naloxone prescribed in the last 12 months (18 non-responses)**				
Yes	1017 (70)	173 (17)		
No	431 (30)	64 (15)	1.1	0.309
***Harms***				
**HIV infection**				
Yes	42 (3)	17 (40)		
No	1332 (97)	210 (16)	17.6	<0.001
**Current HCV infection**				
Yes	402 (32)	99 (25)		
No	840 (68)	111 (13)	25.2	<0.001
**Overdosed in the last year (32 non-responses)**				
Yes	264 (18)	78 (30)		
No	1170 (82)	155 (13)	42.1	<0.001
**Skin and soft tissue infection in the last year (13 non-responses)**				
Yes	401 (28)	87 (22)		
No	1052 (72)	150 (14)	11.8	0.001

a Total may not add up due to missing data.

b Missing values excluded from analysis.

c Non-responses/missing data is only presented if it is greater than 5% of the total sample.

d Excluding Glasgow city centre.

e Excluding NHS Greater Glasgow and Clyde.

**Table 2:** Odds ratios (OR), adjusted odds ratios (aOR) and 95% confidence intervals (CI) of combined demographic/environmental and individual factors associated with reporting public injecting in the last 6 months in Scotland, 2017–18.

	Total^a^^,^ N	Reported public injecting in last 6 months^b^ (% of N)	Overall sample (*n* = 1464, 240 reported public injecting)			
			OR (95% CI)	*p*-value	aOR^e^ (95% CI)	*p*-value
**Age (per year increase)**	39.5	38.6	0.98 (0.96 to 0.99)	0.030	0.97 (0.95 to 0.99)	0.013
**Homeless in last 6 months**						
No	1061	91 (9)	1	<0.001	1	<0.001
Yes	401	148 (37)	6.24 (4.64 to 8.38)		3.68 (2.61 to 5.19)	
**Number of times in prison since first injected drugs**						
Low number (5 incarcerations or less)	911	119 (13)	1	<0.001	1	0.102
High number (more than 5 incarcerations)	542	119 (22)	1.87 (1.41 to 2.47)		1.33 (0.94 to 1.88)	
**Received OST in last 6 months**						
No	217	66 (30)	1	<0.001	1	<0.001
Yes	1247	174 (14)	0.37 (0.27 to 0.51)		0.37 (0.24 to 0.56)	
**Injected cocaine in last 6 months**						
No	1010	111 (11)	1	<0.001	1	0.046
Yes	452	129 (29)	3.23 (2.44 to 4.29)		1.46 (1.00 to 2.13)	
**Alcohol consumption (>14 units per week) in the last year**						
No	1133	137 (12)	1	<0.001	1	<0.001
Yes	317	100 (32)	3.35 (2.49 to 4.51)		2.42 (1.69 to 3.44)	
**Average injection frequency in last 6 months**						
Low frequency (4 times per day or less)	1345	186 (14)	1	<0.001	1	<0.001
High frequency (4 or more times per day)	117	54 (46)	5.34 (3.59 to 7.92)		3.16 (1.93 to 5.18)	
**Recruitment region**						
Rest of Scotland^c^	880	87 (10)	1		1	
NHS Greater Glasgow and Clyde^d^	365	51 (14)	1.48 (1.02 to 2.14)	0.037	2.25 (1.46 to 3.47)	<0.001
Glasgow city centre	219	102 (47)	7.95 (5.62 to 11.22)	<0.001	5.45 (3.48 to 8.54)	<0.001

a Excludes missing data.

b May not add up to 240 PWID due to missing data.

c Excluding NHS Greater Glasgow and Clyde.

d Excluding Glasgow city centre.

e Adjusted for all co-variates presented in the table.

### Prevalence of public injecting

Prevalence of public injecting among the overall sample was 16% (*n* = 240), with highest rates in Glasgow city centre (47%, *n* = 102, *p*<0.001) (Table 1). The prevalence of public injecting in other recruitment areas and the injecting locations utilised can be viewed in the Appendix 7 and 8. The prevalence of public injecting among those reporting homelessness, cocaine injecting and a high injection frequency (>4 times per day), was 37% (*n* = 148, *p*<0.001), 28% (*n* = 129, *p*<0.001) and 46% (*n* = 54, *p*<0.001), respectively. There was a lower prevalence among those reporting receiving OST (14%, *n* = 174, *p*<0.001).

With regard to harms, the prevalence of public injecting among those with HIV infection and active HCV infection was 40% (*n* = 17, *p*<0.001) and 25% (*n* = 99, *p*<0.001), respectively. Among those who reported overdose and SSTI, 30% (*n* = 78, *p*<0.001) and 22% (*n* = 87, *p*<0.001) reported public injecting, respectively (Table 1).

### Factors associated with public injecting

In our combined model (Table 2), public injecting was strongly associated with recruitment region (*p*<0.001), with those recruited in Glasgow city centre with nearly five and a half times the odds (aOR=5.45, 95% CI 3.48 to 8.54) of reporting public injecting compared to participants from the rest of Scotland. Public injecting was also strongly associated with homelessness (aOR=3.68, 95% CI 2.61 to 5.19, *p*<0.001), alcohol consumption (>14 units per week) (aOR=2.42, 95% CI 1.69 to 3.44, *p*<0.001), high injection frequency (≥4 times per day) (aOR=3.16, 95% CI 1.93 to 5.18, *p*<0.001) and cocaine injecting (aOR=1.46, 95% CI 1.00 to 2.13, *p* = 0.046). Receipt of OST (aOR=0.37, 95% CI 0.24 to 0.56, *p*<0.001) and older age (per year increase) (aOR=0.97, 95% CI 0.95 to 0.99, *p* = 0.013) were associated with lower odds of public injecting.

### Harms associated with public injecting

#### HIV infection

Prevalence of HIV infection among those who reported public injecting was 7% (*n* = 17). PWID who reported public injecting had twice the odds (aOR=2.11, 95% CI 1.13 to 3.92, *p* = 0.019) of being infected with HIV (Table 3a).

**Table 3:** Odds ratios (OR), adjusted odds ratios (aOR) and 95% confidence intervals (CI) of harms (HIV, current HCV, overdose and SSTI) when reporting public injecting in the last 6 months in Scotland, 2017–18.

**a) HIV risk model**^c^**^,^**^d^	**Total**^a^**^,^ N**	**HIV infection (% of N)**^b^	**HIV infection (*n*** **=** **1375; 42 positive)**			
			**OR (95% CI)**	**p-value**	**aOR (95% CI)**	**p-value**

**Reported public injecting**						
No	1135	25 (2)	1	<0.001	1	0.019
Yes	227	17 (7)	3.59 (1.91 to 6.77)		2.11 (1.13 to 3.92)	
**b) Current HCV risk model**^c^**^,^**^e^	**Total**^a^**^,^ N**	**Current HCV infection (% of N)**^b^	**Current HCV infection (*n*** **=** **1376; 804 positive)**			
			**OR (95% CI)**	**p-value**	**aOR (95% CI)**	**p-value**

**Reported public injecting**						
No	1032	303 (29)	1	<0.001	1	0.043
Yes	210	99 (47)	2.14 (1.58 to 2.91)		1.49 (1.01 to 2.19)	
**c) Overdose risk model**^c^**^,^**^f^	**Total**^a^**^,^ N**	**Overdosed in last year (% of N)**^b^	**Overdosed in last year (*n*** **=** **1437; 265 overdose)**			
			**OR (95% CI)**	**p-value**	**aOR (95% CI)**	**p-value**

**Reported public injecting**						
No	1201	186 (15)	1	<0.001	1	<0.001
Yes	233	78 (33)	2.75 (2.01 to 3.76)		1.59 (1.27 to 2.01)	
**d) SSTI risk model**^c^**^,^**^g^	**Total**^a^**^,^ N**	**SSTI in the last year (% of N)**^b^	**SSTI in last year (*n*** **=** **1456; 402 SST)**			
			**OR (95% CI)**	**p-value**	**aOR (95% CI)**	**p-value**

**Reported public injecting**						
No	1216	314 (26)	1	0.001	1	<0.001
Yes	237	87 (37)	1.67 (1.24 to 2.23)		1.42 (1.17 to 1.73)	

a Excludes missing data.

b May not add up due to missing data.

c A multi-level framework was applied to each model to adjust for recruitment region, across the 11 NHS board areas. To account for clustering as a result of the HIV outbreak in Glasgow, for the HIV model the multi-level framework was collapsed into NHS Greater Glasgow and Clyde vs. Rest of Scotland.

d Adjusted for age, sex, homelessness and cocaine injecting.

e Adjusted for age, sex, homelessness, cocaine injecting, injection frequency and alcohol consumption (>14 units per week).

f Adjusted for age, sex, homelessness, cocaine injecting, injection frequency and alcohol consumption (>14 units per week).

g Adjusted for age, sex, homelessness, injecting heroin and cocaine at the same time and injection frequency.

#### Current HCV infection

Prevalence of current HCV infection was 47% (*n* = 99) among PWID who reported public injecting. Those who reported public injecting had one and a half times the odds of a current HCV infection than PWID who did not (aOR=1.49, 95% CI, 1.01 to 2.19, *p* = 0.043) (Table 3b).

#### Overdose

Prevalence of self-reported overdose in the last year was 33% (*n* = 78) among PWID who reported public injecting. PWID who reported public injecting had just over one and a half times the odds of reporting an overdose in the last year (aOR=1.59, 95% CI, 1.27 to 2.01, *p*<0.001) (Table 3c).

#### Skin and soft tissue infection (SSTI)

Prevalence of self-reported SSTI infection in the last year was 37% (*n* = 87) among PWID who reported public injecting. We found public injecting was significantly associated with SSTI (aOR=1.42, 95% CI, 1.17 to 1.73, *p*<0.001) (Table 3d).

## Discussion

In the context of one of the most persistent recent outbreaks of HIV infection among PWID internationally (Des Jarlais et al., 2018) and the largest the UK has seen in 30 years, we have examined the extent of public injecting and associated harms in Scotland. We found that 16% of PWID across Scotland, and 47% in Glasgow city centre, reported public injecting. In multivariate analysis, the environmental factors which were most strongly associated with an increased risk of public injecting were recent experience of homelessness and study recruitment in Glasgow city centre (i.e. the epicentre of the HIV outbreak). With regard to individual risk factors, PWID reporting alcohol consumption above recommended guidelines (>14 units per week), a high injection frequency (≥4 times per day) and cocaine injecting had a significantly higher odds of reporting public injecting. We also found that public injecting was strongly associated with HIV infection, current HCV infection, and self-reported overdose and SSTI in the last year.

Our findings support the hypothesis that public injecting was a key risk factor in the HIV outbreak in Glasgow (Tweed et al., 2018). As we have measured the prevalence of HIV, rather than incidence, we cannot deduce when individuals in the survey acquired their HIV infection. However, it is likely that this occurred in the recent past (previous 3 years) in the context of the HIV outbreak and historical low prevalence of HIV infection in previous NESI surveys (McAuley et al., 2019). Before 2017–18, the prevalence of HIV infection in this population was very low; 1% during 2011–14 (1% in Glasgow city centre), rising to 2% in 2017–18 (11% in Glasgow city centre) (Health Protection Scotland, 2019a; McAuley et al., 2019). Previous studies have found an association between public injecting and risk behaviours (e.g. sharing of injecting equipment) that can lead to HIV acquisition (Hunter et al., 2018; Marshall et al., 2010; Mazhnaya et al., 2018; McKnight et al., 2007; Navarro & Leonard, 2004; Vallance et al., 2018; Wood et al., 2001). While the mechanism by which HIV transmission occurs is likely due to the increased risk of sharing of injecting equipment as a result of the complex environmental conditions (Small et al., 2007), this is the first time that the practice of public injecting has been found to be associated with HIV infection based on serological evidence, in the context of a comprehensive harm reduction environment (McAuley et al., 2019). A previous small study conducted in 2001–2002 in Montreal which reported on serological evidence of HIV did not find an association, likely due to a lack of power (Green, Hankins, Palmer, Boivin & Platt, 2003). Furthermore, consistent with previous research conducted in the USA pre HAART, we have demonstrated that public injecting is an important risk factor in cities with a historically low prevalence of HIV (Friedman et al., 1995). Our findings have illuminated the potential role of public injecting, in addition to cocaine injecting and homelessness (which were already considered important drivers of HIV in Glasgow) (McAuley et al., 2019), in facilitating HIV transmission amongst connected injection networks of PWID (McAuley et al., 2019; Ragonnet-Cronin et al., 2018; Sypsa, 2019). Despite a multi-pronged response, including expansion of HIV testing, adoption of treatment as prevention approach through a nurse-led outreach and community prescribing model and needle and syringe provision (Metcalfe, Glover, Patton, Brown & Peters, 2018), this outbreak has persisted in contrast to other recent outbreaks of HIV amongst PWID internationally (Des Jarlais et al., 2018), suggesting that more needs to be done to address the wider determinants of HIV transmission in this vulnerable population of PWID, such as public injecting.

In addition to HIV infection, we also found an independent association between public injecting and current HCV infection. The prevalence of HCV among PWID is known to vary across Scotland, with highest rates reported for Glasgow (Health Protection Scotland, 2019a). Even considering those recruited from Glasgow city, the prevalence of current HCV infection among PWID who reported public injecting (47%) was higher than those who had not reported public injecting (29%). The acquisition of HCV infection may have been acquired at any time in the past, given the historically high prevalence of HCV in Scotland (Health Protection Scotland, 2019a). Our results suggest that PWID (infected with HCV) are engaging with high risk injection practices, which increases the risk of onward transmission and re-infection among those treated. This is an important finding as could pose a threat to efforts to eliminate HCV infection – both locally in Glasgow and nationally in Scotland – in advance of the WHO 2030 global elimination date (Health Protection Scotland, 2019b; World Health Organisation, 2016).

Though the relationship between public injecting and overdose has been well demonstrated, particularly in North America (Hunter et al., 2018; Sutter et al., 2019; Vallance et al., 2018; Wallace et al., 2019), to the best of our knowledge this is the first study which has demonstrated an association between overdose and public injecting in the UK. This has important implications and suggests that public injecting may be contributing to Scotland's record levels of drug-related deaths (DRD) (National Records of Scotland, 2019). Ethnographic research has demonstrated that the social context in which public injecting takes place increases the risk of overdose due to PWID fear of arrest, assault or discovery by the public. This unregulated environment causes rushed, higher risk injections, in locations where PWID may not be discovered immediately in the event of an overdose (Darke et al., 2001; Small et al., 2007). The contexts of the high rates of DRD and overdose between the UK and North America are distinct, with North America experiencing a triple-wave epidemic driven initially by over-prescribing prescription opioids, then by heroin, and subsequently by extremely potent synthetic opioids such as fentanyl (Ciccarone, 2019). In contrast, the situation in Scotland is different, with increasing DRD partly related to an ageing population of PWID with complex health needs consuming multiple drugs in combination, typically heroin, benzodiazepines and alcohol (National Records of Scotland, 2019). However, what is highlighted in both settings is that the status quo harm reduction response is no longer sufficient and new policies and interventions must be considered to prevent these avoidable deaths.

Our findings are consistent with previous research demonstrating a link between people who inject in public and SSTI (Salmon et al., 2009), as a result of their highly unsanitary injecting environment (Dunleavy, Hope, Roy & Taylor, 2019; Small et al., 2007) and the reduced likelihood of following injecting related hygiene practices (Marshall et al., 2010). In recent years, Scotland has experienced major outbreaks of spore-forming bacteria, including an outbreak of anthrax (Palmateer, Ramsay, Browning, Goldberg & Hutchinson, 2012) and the largest documented outbreak of wound botulism in Europe (Trayner et al., 2018), both associated with a contaminated drug supply. In both outbreaks, the majority of cases presented with SSTI (Palmateer et al., 2012) and were engaged in high risk injection practices such as muscle/skin popping (a risk factor for SSTI) (Trayner et al., 2018). Public injecting was not quantified in these previous outbreaks, but the environmental conditions created by public injecting may have been a contributing factor and certainly warrant further investigation.

Compared to “open drug scenes”, like the well-documented Downtown Eastside in Vancouver (Small et al., 2007), public injecting in the UK has been considered much less salient. However, in areas like Glasgow, public injecting is often not visible to the general public, despite being close to busy city centre restaurant and shopping areas (Tweed et al., 2018). A needs assessment focusing on the health needs of those injecting in public places in Glasgow was conducted in 2016 (Tweed et al., 2018), which estimated that there are 400–500 PWID regularly injecting in public places in Glasgow city centre. The last paper on public injecting in the UK was published in 2007 among a small sample of PWID (*n* = 301) (Hunt et al., 2007), where they found a public injecting prevalence of 55% among needle and syringe service attendees in London and Leeds. While our national prevalence was much lower (16%), rates in Glasgow city centre were comparable (47%) and we found that those who were recruited in Glasgow city centre had nearly a five and a half times higher odds of reporting public injecting when compared to those recruited in the rest of Scotland. This result in particular, should be of acute interest to policy makers in the UK, given the recent rejection by the UK government to establish the UK's first drug consumption room (DCR) in Glasgow city centre, which has received considerable media attention (Atkinson, McAuley, Trayner & Sumnall, 2019). We have provided substantial new evidence to inform the need for such an intervention, by demonstrating a high prevalence of public injecting and its association with drug-related harms.

This study adds to the large body of literature which demonstrates how public injecting and the physical micro-environment in which injecting drug use occurs predisposes individuals to harm (Hunter et al., 2018; Rhodes, 2002, 2009; Small et al., 2007; Vallance et al., 2018). DCRs provide an alternative, clean and safe environment for PWID injecting in public; the evidence base in support of DCRs is substantial (Pardo, Caulkins & Kilmer, 2018; Potier, Laprévote, Dubois-Arber, Cottencin & Rolland, 2014), particularly in regard to reducing the harms associated with public injecting (Potier et al., 2014; Wood et al., 2004; Wood, Tyndall, Montaner & Kerr, 2006). Arguments for their introduction, are particularly strong in areas such as Glasgow, which are currently experiencing significant health crises among PWID (Caulkins, Pardo & Kilmer, 2019; McAuley et al., 2019; Tweed et al., 2018). Further weight could be added to the proposals in Glasgow by assessing the acceptability of a DCR among vulnerable PWID in Scotland, which would provide an indication of both the demand for the service and whether it would be successful at attracting high risk PWID. However, given the multi-faceted nature of public injecting, the introduction of DCRs should not be viewed as a panacea to address all harms within this population. DCRs should be implemented as part of a comprehensive harm reduction approach, including OST, needle exchange, safe injecting advice, naloxone and access to recovery services.

In areas of Scotland, there have been reports of sub-optimal methadone dosing and of poor retention rates among PWID engaging with OST services (House of Commons Scottish Affairs Committee, 2019), despite approximately 70% of current PWID reported receiving OST in the last 6 months (Health Protection Scotland, 2019a). We found that OST was a protective factor against public injecting, which highlights the importance and value of optimising, and where needed, extending current drug treatment services. This includes, Heroin Assisted Treatment (HAT), referring to the prescribing of injectable diamorphine as a treatment for opiate dependence, which has just been made available in Glasgow city centre embedded in a service providing care for those experiencing homelessness. Furthermore, access to injecting equipment provision services (that provide needles/syringes, foil, water, filters. etc.) should also be enhanced, which has been a key response to the HIV outbreak in Glasgow. Delivering harm reduction interventions through outreach programmes, including peer and social network approaches to the delivery of these services, could also further enhance their impact. Furthermore, training and engagement with police on public health approaches to issues with public drug use, has been shown to be effective in encouraging PWID to engage with harm reduction services (Beletsky, Thomas, Shumskaya, Artamonova & Smelyanskaya, 2013; DeBeck et al., 2008).

In addition to established harm reduction interventions, a number of social and structural interventions should be considered to address upstream social risk factors that lead PWID to public injecting, (such as homelessness), including ensuring equitable access to housing and financial benefits (Degenhardt et al., 2010). This should include initiatives, such as Housing First, which consider “housing as harm reduction” to address the consistently overlapping issues of homelessness and substance use (Pauly, Reist, Belle-Isle & Schactman, 2013). They do not require individuals to undergo treatment for their substance use or to be abstinent to access and keep permanent housing; by providing a safe and secure environment they can reduce harms associated with drug use and homelessness (Baxter, Tweed, Katikireddi & Thomson, 2019). A Housing First approach is a key component of the Glasgow City Health and Social Care Partnership (HSCP) rapid rehousing transition plan, which aims to eliminate homelessness in Glasgow within 5 years (Glasgow City Health & Social Care Partnership, 2019). Furthermore, welfare and social security reform should also be considered, including removing the sanctions based approach currently adopted by the UK Government (House of Commons Scottish Affairs Committee, 2019). This approach penalises individuals by reducing welfare payments in response to failure to meet certain commitments (such as failure to attend meetings), which disproportionately affect individuals in precarious living situations (Economic & Social Research Council, 2018). This is supported by a Canadian study, which showed that receiving the most income from welfare was protective against public injecting (Navarro & Leonard, 2004).

This study had a number of limitations which should be acknowledged. Firstly, risk factor data and some outcome data (i.e. overdose and SSTI) collected through NESI is self-reported, which may be subject to response bias. However, this is considered minimal based on our use of independent researchers to collect the data and previous research (Darke, 1998). The sample is also biased towards those who utilised IEP services, and thus may not fully represent the PWID population or PWID who inject in public. However, data presented in the previously described needs assessment (Tweed et al., 2018), highlighted that PWID in Glasgow city centre (with nearly 50% injecting in public) are regular attendees of needle and syringe provisions sites, which suggests that the majority of PWID do attend these services. Although NESI collects data on a wide range of risk factors, residual confounding may remain a factor in our models. Furthermore, temporality cannot be established with certainty in our analyses as some exposure data (such as homelessness and public injecting) refers to the last 6 months and some of our outcomes (such as SSTI and overdose) refer to the last 12 months. Additionally, recall bias may be present given participants were asked to recall behaviours and harms from 6 months to a year.

## Conclusion

To the best of our knowledge, this is the first study to conduct a national assessment of public injecting in the context of a large ongoing HIV outbreak, where we have established, based on serological evidence, that public injecting is independently associated with HIV infection. We have also demonstrated that public injecting is associated with multiple adverse outcomes, including current HCV, overdose and SSTI, highlighting how the complex nature of the public injecting risk environment increases risk of drug-related harm. Given the breadth of these harms, these results highlight the need to address the issue of public injecting, through a combination of interventions, including those which consider how structural and social inequalities influence the risk of harms. Furthermore, we have provided robust epidemiological evidence to support the optimisation and expansion of harm reduction services internationally. Our findings specifically have relevance in the current context in the UK, by providing further weight to proposals to establish the UK's first DCR in Glasgow and reinforce the need for better harm reduction policies in the UK.

## Conflict of interest statement

SJH has received honoraria from Gilead, unrelated to this study. All remaining authors have nothing to disclose.

## Appendix Supplementary materials

Image, application 1
