# Supplementary material for: Increased risk of HIV and other drug-related harms associated with injecting in public places: national bio-behavioural survey of people who inject drugs
Source: Int J Drug Policy. 2020 Mar;77:102663. doi: 10.1016/j.drugpo.2020.102663 (PMC8330401; doi:10.1016/j.drugpo.2020.102663)
Supplement: Supplementary file 1 [file mmc1.docx]

| **Appendix 1. Odds ratios (OR), adjusted odds ratios (aOR) and 95% confidence intervals (CI) of demographic/environmental factors associated with reporting public injecting in the last 6 months in Scotland, 2017-18** | | | | | | |
| --- | --- | --- | --- | --- | --- | --- |
|  | **Total^a,^ N** | **Reported public injecting in last 6 months (% of N)^b^** | **Overall sample (n=1464, 240 reported public injecting)** | | | |
|  |  |  | **OR (95% CI)** | **p-value** | **aOR^e^ (95% CI)** | **p-value** |
| **Gender** |  |  |  |  |  |  |
| Male | 1095 | 196 (18) | 1 | 0.006 | 1 | 0.255 |
| Female | 366 | 43 (12) | 0.61 (0.42 to 0.87) |  | 0.76 (0.48 to 1.22) |  |
| **Age (per year increase)** | 39.5 | 38.6 | 0.98 (0.96 to 0.99) | 0.030 | 0.96 (0.94 to 0.99) | 0.009 |
| **Homeless in last 6 months** |  |  |  |  |  |  |
| No | 1061 | 91 (9) | 1 | <0.001 | 1 | <0.001 |
| Yes | 401 | 148 (37) | 6.24 (4.64 to 8.38) |  | 4.71 (3.24 to 6.82) |  |
| **Number of times in prison since first injected drugs** | |  |  |  |  |  |
| Low number (5 incarcerations or less) | 911 | 119 (13) | 1 | <0.001 | 1 | 0.042 |
| High number (more than 5 incarcerations) | 542 | 119 (22) | 1.87 (1.41 to 2.47) |  | 1.47 (1.02 to 2.15) |  |
| **Received OST in the last 6 months** |  |  |  |  |  |  |
| No | 217 | 66 (30) | 1 | <0.001 | 1 | <0.001 |
| Yes | 1247 | 174 (14) | 0.37 (0.27 to 0.51) |  | 0.28 (0.18 to 0.44) |  |
| **Syringe coverage per injecting episode in last 6 months** |  |  |  |  |  |  |
| 100%+ | 1016 | 149 (15) | 1 | 0.153 | 1 | 0.493 |
| <100% | 281 | 51 (18) | 1.29 (0.91 to 1.83) |  | 1.16 (0.76 to 1.77) |  |
| **Arrested for drug offenses in the last 6 months** | |  |  |  |  |  |
| No | 1245 | 193 (16) | 1 | 0.067 | 1 | 1.00 |
| Yes | 182 | 38 (21) | 1.44 (0.97 to 2.12) |  | 1.00 (0.61 to 1.65) |  |
| **Recruitment region** |  |  |  |  |  |  |
| Rest of Scotland^c^ | 880 | 87 (10) | 1 |  | 1 |  |
| NHS Greater Glasgow and Clyde^d^ | 365 | 51 (14) | 1.48 (1.02 to 2.14) | 0.037 | 2.33 (1.41 to 3.87) | 0.001 |
| Glasgow city centre | 219 | 102 (47) | 7.95 (5.62 to 11.22) | <0.001 | 7.76 (4.99 to 12.05) | <0.001 |
|  |  |  |  |  |  |  |
| ^a^ Excludes missing data |  |  |  |  |  |  |
| ^b^ May not add up to 240 PWID due to missing data | |  |  |  |  |  |
| ^c^ Excluding NHS Greater Glasgow and Clyde | |  |  |  |  |  |
| ^d^ Excluding Glasgow city centre |  |  |  |  |  |  |
| ^e^ Adjusted for all co-variates presented in the table | |  |  |  |  |  |

| **Appendix 2. Odds ratios (OR), adjusted odds ratios (aOR) and 95% confidence intervals (CI) of individual factors associated with reporting public injecting in the last 6 months in Scotland, 2017-18** | | | | | | |
| --- | --- | --- | --- | --- | --- | --- |
|  |  |  |  |  |  |  |
|  | **Total^a,^ N** | **Reported public injecting in last 6 months (% of N)^b^** | **Overall sample (n=1464, 240 public injectors)** | | | |
|  |  |  | **OR (95% CI)** | **p-value** | **aOR^c^ (95% CI)** | **p-value** |
| **Injected cocaine in last 6 months** |  |  |  |  |  |  |
| No | 1010 | 111 (11) | 1 | <0.001 | 1 | <0.001 |
| Yes | 452 | 129 (29) | 3.23 (2.44 to 4.29) |  | 2.66 (1.97 to 3.61) |  |
| **Shared needles/syringes in last 6 months** |  |  |  |  |  |  |
| No | 1311 | 201 (15) | 1 | 0.008 | 1 | 0.080 |
| Yes | 141 | 34 (24) | 1.75 (1.16 to 2.65) |  | 1.49 (0.95 to 2.35) |  |
| **Alcohol consumption (>14 units per week) in the last year** | | |  |  |  |  |
| No | 1133 | 137 (12) | 1 | <0.001 | 1 | <0.001 |
| Yes | 317 | 100 (32) | 3.35 (2.49 to 4.51) |  | 2.99 (2.18 to 4.11) |  |
| **Average injection frequency in last 6 months** | |  |  |  |  |  |
| Low frequency (4 times per day or less) | 1345 | 186 (14) | 1 | <0.001 | 1 | <0.001 |
| High frequency (4 or more times per day) | 117 | 54 (46) | 5.34 (3.59 to 7.92) |  | 4.44 (2.89 to 6.82) |  |
|  |  |  |  |  |  |  |
| ^a^ Excludes missing data |  |  |  |  |  |  |
| ^b^ May not add up to 240 PWID due to missing data | |  |  |  |  |  |
| ^c^ Adjusted for all co-variates presented in the table | |  |  |  |  |  |

| **Appendix 3. Odds ratios (OR), adjusted odds ratios (aOR) and 95% confidence intervals (CI) of HIV infection when reporting public injecting in the last 6 months in Scotland, 2017-18** | | | | | | |
| --- | --- | --- | --- | --- | --- | --- |
|  |  |  |  |  |  |  |
|  | **Total^a,^ N** | **HIV infection (% of N)^b^** | **HIV infection (n=1375; 42 positive)** | | | |
|  |  |  | **OR (95% CI)** | **p-value** | **aOR (95% CI)** | **p-value** |
| **Reported public injecting** |  |  |  |  |  |  |
| No | 1135 | 25 (2) | 1 | <0.001 | 1 | 0.019 |
| Yes | 227 | 17 (7) | 3.59 (1.91 to 6.77) |  | 2.11 (1.13 to 3.92) |  |
| **Age (per year increase)** | 39.5 | 41.5 | 1.04 (0.99 to 1.08) | 0.081 | 1.05 (0.97 to 1.15) | 0.184 |
| **Gender** |  |  |  |  |  |  |
| Male | 1027 | 26 (3) | 1 | 0.043 | 1 | <0.001 |
| Female | 336 | 16 (5) | 1.92 (1.01 to 3.63) |  | 3.08 (2.38 to 4.01) |  |
| **Homeless in last 6 months** |  |  |  |  |  |  |
| No | 991 | 20 (2) | 1 | <0.001 | 1 | <0.001 |
| Yes | 374 | 22 (6) | 3.03 (1.63 to 5.62) |  | 1.75 (1.65 to 1.85) |  |
| **Injected cocaine in the last 6 months** |  |  |  |  |  |  |
| No | 941 | 10 (1) | 1 | <0.001 | 1 | <0.001 |
| Yes | 421 | 32 (8) | 7.66 (3.72 to 15.73) |  | 6.44 (5.42 to 7.66) |  |
|  |  |  |  |  |  |  |
| ^a^ Excludes missing data |  |  |  |  |  |  |
| ^b^ May not add up to 42 PWID due to missing data | |  |  |  |  |  |

| **Appendix 4. Odds ratios (OR), adjusted odds ratios (aOR) and 95% confidence intervals (CI) of current HCV infection when reporting public injecting in the last 6 months in Scotland, 2017-18** | | | | | | |
| --- | --- | --- | --- | --- | --- | --- |
|  |  |  |  |  |  |  |
|  | **Total^a,^ N** | **Active HCV^b^ (% of N)** | **Current HCV infection (n=1255; 402 positive)** | | | |
|  |  |  | **OR (95% CI)** | **p-value** | **aOR (95% CI)** | **p-value** |
| **Reported public injecting** |  |  |  |  |  |  |
| No | 1032 | 303 (29) | 1 | <0.001 | 1 | 0.043 |
| Yes | 210 | 99 (47) | 2.14 (1.58 to 2.91) |  | 1.49 (1.01 to 2.19) |  |
| **Age (per year increase)** | 39.5 | 40.3 | 1.01 (1.00 to 1.03) | 0.027 | 1.02 (1.01 to 1.04) | 0.026 |
| **Gender** |  |  |  |  |  |  |
| Male | 937 | 311 (33) | 1 | 0.263 | 1 | 0.856 |
| Female | 306 | 91 (30) | 0.85 (0.64 to 1.13) |  | 1.02 (0.83 to 1.25) |  |
| **Homeless in last 6 months** |  |  |  |  |  |  |
| No | 902 | 257 (29) | 1 | <0.001 | 1 | 0.010 |
| Yes | 344 | 146 (42) | 1.85 (1.42 to 2.40) |  | 1.39 (1.08 to 1.79) |  |
| **Injected cocaine in the last 6 months** |  |  |  |  |  |  |
| No | 868 | 240 (28) | 1 | <0.001 | 1 | 0.043 |
| Yes | 374 | 162 (43) | 1.99 (1.55 to 2.57) |  | 1.67 (1.02 to 2.75) |  |
| **Average injection frequency in last 6 months** |  |  |  |  |  |  |
| Low frequency (4 times per day or less) | 1138 | 358 (31) | 1 | 0.034 | 1 | 0.533 |
| High frequency (4 or more times per day) | 103 | 43 (42) | 1.56 (1.04 to 2.36) |  | 1.08 (0.85 to 1.38) |  |
| **Alcohol consumption (>14 units per week) in the last year** | | |  |  |  |  |
| No | 964 | 279 (29) | 1 | <0.001 | 1 | 0.001 |
| Yes | 271 | 122 (45) | 2.01 (1.52 to 2.66) |  | 1.62 (1.23 to 2.14) |  |
|  |  |  |  |  |  |  |
| ^a^ Excludes missing data |  |  |  |  |  |  |
| ^b^ May not add up to 402 PWID due to missing data | |  |  |  |  |  |

| **Appendix 5. Odds ratios (OR), adjusted odds ratios (aOR) and 95% confidence intervals (CI) of overdose in the last year if reporting public injecting in the last 6 months in Scotland, 2017-18.** | | | | | | |
| --- | --- | --- | --- | --- | --- | --- |
|  |  |  |  |  |  |  |
|  | **Total^a,^ N** | **Overdosed in last year (% of N)^b^** | **Overdosed in last year (n=1437; 265 overdose)** | | | |
|  |  |  | **OR (95% CI)** | **p-value** | **aOR* (95% CI)** | **p-value** |
| **Reported public injecting** |  |  |  |  |  |  |
| No | 1201 | 186 (15) | 1 | <0.001 | 1 | <0.001 |
| Yes | 233 | 78 (33) | 2.75 (2.01 to 3.76) |  | 1.59 (1.27 to 2.01) |  |
| **Age (per year increase)** | 39.5 | 38.3 | 0.97 (0.95 to 0.99) | 0.001 | 0.97 (0.94 to 0.99) | 0.014 |
| **Gender** |  |  |  |  |  |  |
| Male | 1077 | 206 (19) | 1 | 0.273 | 1 | 0.644 |
| Female | 357 | 59 (17) | 0.84 (0.61 to 1.15) |  | 0.89 (0.54 to 1.46) |  |
| **Homeless in last 6 months** |  |  |  |  |  |  |
| No | 1041 | 152 (15) | 1 | <0.001 | 1 | <0.001 |
| Yes | 394 | 113 (29) | 2.35 (1.78 to 3.11) |  | 1.73 (1.34 to 2.24) |  |
| **Injected cocaine in the last 6 months** |  |  |  |  |  |  |
| No | 987 | 148 (15) | 1 | <0.001 | 1 | <0.001 |
| Yes | 446 | 115 (26) | 1.97 (1.49 to 2.59) |  | 1.45 (1.22 to 1.72) |  |
| **Average injection frequency in last 6 months** | |  |  |  |  |  |
| Low frequency (4 times per day or less) | 1318 | 225 (17) | 1 | <0.001 | 1 | 0.089 |
| High frequency (4 or more times per day) | 115 | 39 (34) | 2.49 (1.65 to 3.76) |  | 1.63 (0.92 to 2.85) |  |
| **Alcohol consumption (>14 units per week) in the last year** | | |  |  |  |  |
| No | 1109 | 176 (16) | 1 | <0.001 | 1 | <0.001 |
| Yes | 314 | 87 (28) | 2.03 (1.51 to 2.71) |  | 1.63 (1.31 to 2.02) |  |
|  |  |  |  |  |  |  |
| ^a^ Excludes missing data |  |  |  |  |  |  |
| ^b^ May not add up to 265 PWID due to missing data | |  |  |  |  |  |

| **Appendix 6. Odds ratios (OR), adjusted odds ratios (aOR) and 95% confidence intervals (CI) of skin and soft tissue infection (SSTI) in the last year if reporting public injecting in the last 6 months in Scotland, 2017-18.** | | | | | | |
| --- | --- | --- | --- | --- | --- | --- |
|  |  |  |  |  |  |  |
|  | **Total^a,^ N** | **SSTI in the last year (% of N)^b^** | **SSTI in last year (n=1456; 402 SSTI)** | | | |
|  |  |  | **OR (95% CI)** | **p-value** | **aOR* (95% CI)** | **p-value** |
| **Reported public injecting** |  |  |  |  |  |  |
| No | 1216 | 314 (26) | 1 | 0.001 | 1 | <0.001 |
| Yes | 237 | 87 (37) | 1.67 (1.24 to 2.23) |  | 1.42 (1.17 to 1.73) |  |
| **Age (per year increase)** | 39.5 | 40.2 | 1.02 (0.99 to 1.03) | 0.053 | 1.01 (1.02 to 1.03) | <0.001 |
| **Gender** |  |  |  |  |  |  |
| Male | 1093 | 292 (27) | 1 | 0.151 | 1 | 0.007 |
| Female | 359 | 110 (31) | 1.21 (0.93 to 1.57) |  | 1.42 (1.11 to 1.83) |  |
| **Homeless in last 6 months** |  |  |  |  |  |  |
| No | 1056 | 272 (26) | 1 | 0.012 | 1 | 0.197 |
| Yes | 398 | 129 (32) | 1.38 (1.08 to 1.78) |  | 1.20 (0.91 to 1.59) |  |
| **Injecting heroin and cocaine at the same time** | |  |  |  |  |  |
| No | 1326 | 350 (26) | 1 | 0.001 | 1 | 0.004 |
| Yes | 126 | 51 (41) | 1.89 (1.31 to 2.76) |  | 1.68 (1.18 to 2.39) |  |
| **Average injection frequency in last 6 months** | |  |  |  |  |  |
| Low frequency (4 times per day or less) | 1337 | 352 (26) | 1 | <0.001 | 1 | 0.009 |
| High frequency (4 or more times per day) | 115 | 49 (43) | 2.07 (1.41 to 3.07) |  | 1.74 to (1.15 to 2.64) |  |
|  |  |  |  |  |  |  |
| ^a^ Excludes missing data |  |  |  |  |  |  |
| ^b^ May not add up to 402 PWID due to missing data | |  |  |  |  |  |

| **Appendix 7: Prevalence of public injecting in Scotland by recruitment region, among 1469 PWID who reported injecting in the last 6 months, 2017-18.** | | |
| --- | --- | --- |
|  |  |  |
| **Recruitment region** | **Total sample^a,^ N (%)** | **PWID who reported public injecting (% of N)** |
| Total | 1464 | 240 (16) |
| NHS Greater Glasgow and Clyde | 584 | 153 (26) |
| NHS Lothian | 151 | 30 (20) |
| NHS Lanarkshire | 124 | 13 (10) |
| NHS Ayrshire and Arran | 122 | 9 (7) |
| NHS Forth Valley | 79 | 5 (6) |
| NHS Fife | 64 | 5 (8) |
| NHS Tayside | 146 | 21 (14) |
| NHS Grampian | 101 | 3 (3) |
| NHS Highland | 29 | 0 |
| NHS Dumfries and Galloway | 39 | 1 (3) |
| NHS Borders | 25 | 0 |
|  |  |  |
| ^a^ Excludes missing data |  |  |

| **Appendix 8: Injecting locations among the 240 PWID who reported public injecting in the last 6 months, 2017-18** | | | | | | | |
| --- | --- | --- | --- | --- | --- | --- | --- |
|  |  |  |  |  |  |  |  |
| **Injecting location** | **N (%)** |  |  |  |  |  |  |
| Total | 240 (100) |  |  |  |  |  |  |
| Public toilet | 127 (53) |  |  |  |  |  |  |
| Car park | 80 (33) |  |  |  |  |  |  |
| Stairwell/close | 135 (56) |  |  |  |  |  |  |
| Outdoors | 127 (52.9) |  |  |  |  |  |  |
| Squat/abandoned house | 35 (15) |  |  |  |  |  |  |
| Other | 32 (13) |  |  |  |  |  |  |
